# Angiotensin‐1‐converting enzyme inhibition, antioxidant activity, and modulation of cerebral Na+/K+ ATPase by free phenolics of African locust bean (Parkia biglobosa)

**DOI:** 10.1002/hsr2.17

**Published:** 2017-11-27

**Authors:** Kayode Komolafe, Afolabi C. Akinmoladun, Titilope R. Komolafe, Mary T. Olaleye, Aline A. Boligon, Akintunde A. Akindahunsi, Joao B.T. Rocha

**Affiliations:** ^1^ Department of Biochemistry, School of Sciences The Federal University of Technology Akure Nigeria; ^2^ Department of Biochemistry, Faculty of Science Federal University Oye‐Ekiti Oye‐Ekiti Nigeria; ^3^ Department of Biochemistry and Molecular Biology, CCNE Federal University of Santa Maria Santa Maria RS Brazil

**Keywords:** angiotensin‐converting enzyme, hypotensive, P biglobosa, phenolics

## Abstract

**Aims:**

To investigate the antioxidant activities and effects of free phenols (FPPB) and bound phenols (BPPB) of Parkia biglobosa leaves on some enzymes of neuro‐cardiovascular relevance.

**Methods and Results:**

HPLC‐DAD fingerprinting of FPPB and BPPB, and the antihemolytic, radical (1,1‐diphenyl‐2 picrylhydrazyl, DPPH; 2,2‐azino‐bis(3‐ethylbenzthiazoline‐6‐sulphonic acid), ABTS) scavenging and ferric reducing antioxidant properties of extracts, were assessed. In addition, the effects of the phenolics on angiotensin‐1‐converting enzyme (ACE), cerebral acetylcholinesterase/butyrylcholinesterase (AChE/BuChE), and Na^+^/K^+^ATPase were determined in vitro. FPPB was more potent than BPPB in terms of ABTS (EC_50_:4.06 ± 0.3 vs 24.07 ± 2.1 μg/mL) and DPPH (EC_50_:3.82 ± 0.2 vs 10.22 ± 0.1 μg/mL) radicals scavenged, respectively. The free phenolic extract was a better DPPH^.^ scavenger than ascorbic acid (EC_50_ = 12.58 ± 0.4 μg/mL; DPPH reference) and compared well with Trolox (EC_50_:4.44 ± 0.08 μg/mL; ABTS reference). The anti‐hemolytic effect of FPPB (36%) and BPPB (53%) was highest at 15 μg/mL but lower than that recorded for ascorbic acid (67% at 10 μg/mL). Even though FPPB (IC_50_ = 15.35 ± 4.0 μg/mL) and BPPB (IC_50_ = 46.85 ± 3.3 μg/mL) showed considerably lower ACE‐inhibitory effect than ramipril (IC_50_:0.173 ± 0.04 μg/mL), both extracts demonstrated dose‐dependent, significant (p < 0.01/p < 0.05) inhibition of the enzyme. FPPB increased cerebral Na+/K+ATPase activity but neither phenolic extract affects cerebral AChE/BuChE activities. HPLC‐DAD revealed catechin, caffeic acid, and quercetin, respectively, as the major phenolics (mg/g) in FPPB (29.85, 30.29, and 17.10) and BPPB (32.70, 30.51, and 19.25).

**Conclusion:**

The effects of P biglobosa on ACE and cerebral ATPase are related to its constituent phenolics. ACE inhibition could be an important mechanism underlying the documented hypotensive effect of the plant.

## Abstract 2

Phenolic fingerprinting, antioxidant activities and effects of free phenols (FPPB) and bound phenols (BPPB) of *Parkia biglobosa* leaves on some neuro‐cardiovascular enzymes were evaluated. On the overall, FPPB showed better antioxidant activity than BPPB and compared well with reference standards. FPPB also inhibits angiotensin‐1‐converting enzyme (ACE) and augments cerebral Na+/K+ ATPase. The effects of *P. biglobosa* on the enzymes are thus related to its constituent phenols and the documented hypotensive effect of the plant could proceed via ACE inhibition mechanism.

## 1 INTRODUCTION

Many pathological conditions have a significant relationship with oxidative stress. This reveals the importance of antioxidants because they are proven antidotes to the damaging effects of free radicals which are responsible for oxidative stress.^1^ Advocacy for the use of natural antioxidants in place of synthetic ones is becoming increasingly stronger due to the toxicities associated with the latter.^1^, ^2^ Polyphenols constitute an important group of natural antioxidants in plants that could help prevent or mitigate the effects of oxidative stress on the body. Apart from their roles in attenuating oxidative stress, plant phenolics could also provide other health benefits related to or distinct from their antioxidant property. For instance, the widely documented inhibition of angiotensin‐converting enzyme (ACE) by plant extracts and/or plant‐derived compounds was said to be largely dependent on the phenolic constituents, including flavonoids.^3^, ^4^, ^5^, ^6^ One plausible mechanism of ACE inhibition by phenolics, notably the flavonoids, is the generation of chelates capable of forming complexes within the active centre of ACE thereby inactivating the enzyme.^3^, ^6^

Phenolic compounds exist as either free, solvent extractable form or covalently bound to the plant matrix in plant cells. Extraction of the latter into water or aqueous/organic solvents mixtures is virtually impossible.^7^ It is pertinent to give considerable attention to the bound forms, which constitutes 4% to 57% of total phenolic in plants, to prevent underestimating the antioxidant contents, activities as well as the therapeutic efficacy of different medicinal plants.^8^

Parkia biglobosa (Jacq.) Benth., commonly known as “African locust bean,” is a tropical tree in West Africa popular for its uses as food and medicine. In Southwestern Nigeria, the seeds are fermented to make *Iru*‐ a strong smelling and tasty soup condiment rich in protein.^9^ Ethnomedicinally, the plant has wide applications in the treatment of hypertension and fevers^10^ in tropical Africa. The crude hydromethanolic extract of the leaf was reported to lower blood pressure,^11^ exhibit cardioprotective effect against doxorubicin toxicity^12^ and protect against neurotoxic agents in rat brain hippocampal slices.^13^ Preliminary phytochemical investigation of P biglobosa leaf extract revealed the presence of polyphenols, saponins, cardiac glycosides, terpenoids, and alkaloids,^12^ while further fingerprinting of the phenolics gave caffeic acids, quercetin, and catechin derivatives as the principal antioxidant phytochemicals in the crude extracts (CEs) of the leaf and bark of the plant.^9^, ^13^

The therapeutic mechanisms of P biglobosa are still not well understood, and little or no information is available on the effects of its component phenolics on key enzymes of cardiovascular and neurological relevance. The present study, therefore, sought to compare the antioxidant property of the free and bound phenolics of P biglobosa and evaluate their effects on angiotensin‐1 converting enzyme as well as cerebral acetylcholinesterase, butyrylcholinesterase, and Na+/K+‐ATPase in vitro.

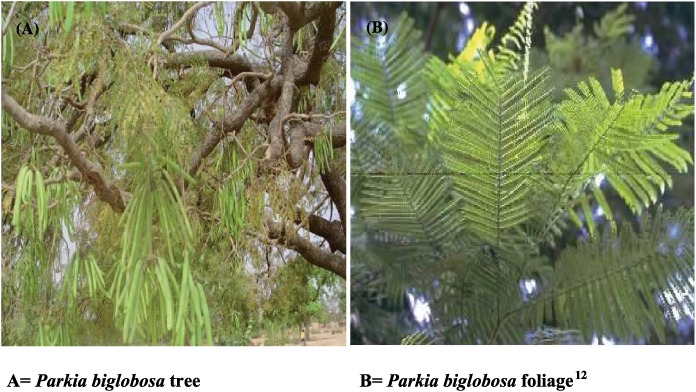

## 2 MATERIALS AND METHODS

### 2.1 Chemicals

2,2‐Diphenlyl‐1‐picrylhydrazyl (DPPH), 2,2′‐azinobis‐(3‐ethylbenzothiazoline‐6‐sulfonic acid) (ABTS) and 6‐hydroxy‐2,5,7,8‐tetramethychroman‐2‐carboxylic acid (Trolox) were obtained from Sigma (St. Louis, MO, USA). Analytical grade phenolic standards (gallic acid, catechin, chlorogenic acid, caffeic acids, epigallocatechin/gallate, rutin, quercetin, kaempferol) and solvents (methanol, acetic acid) were purchased from Merck KGaA, Darmstadt, Germany. Potassium persulfate (K_2_S_2_O_8_), ascorbic acid, and trichloroacetic acid (TCA) were obtained from Sisco Research Laboratories Pvt. Ltd., Mumbai, India. All other reagents and chemicals were of analytical grade.

### 2.2 Plant material

The collection of fresh leaves of P biglobosa was done at a private farm in Isua‐Akoko, Ondo State, Nigeria. P biglobosa leaf was identified and authenticated at the herbarium of the Forestry Research Institute Ibadan, Nigeria where a voucher no (109603) was assigned.

### 2.3 Extraction of free phenolics

#### 2.3.1 Extraction of free phenols of P biglobosa (FPPB)

P biglobosa leaves were kept away from direct sunlight to prevent oxidative damage to the sensitive phytochemicals while air‐drying at room temperature for 28 days. The dried leaves were then ground to fine powder (0.25 to 0.75‐mm particle size) using a blender. The powdered plant material (500 g) was macerated in 1200 mL of 80% methanol for 48 hours. The resulting suspension was filtered and the entire methanol removed using a rotary evaporator. The concentrated extract was thereafter lyophilized to obtain the crude extract (CE). Extraction of the free phenols of P biglobosa leaf (FPBP) was carried out as described by Chu et al.^14^ CE (10 g) was extracted with 80% acetone (1:5 v/v) for 10 hours at room temperature. The mixture was filtered through Whatman no. 2 filter paper on a Büchner funnel under vacuum. The residue obtained (R1) was used for the extraction of bound polyphenols. The filtrate so obtained was evaporated on a rotary evaporator under vacuum at 45°C until approximately 90% of the filtrate has been lost.

#### 2.3.2 Extraction of bound phenols of P biglobosa (BPPB)

R1 was hydrolyzed directly with NaOH (4 M, 20 mL) at room temperature with shaking (1 hour, 40 rpm). Concentrated hydrochloric acid was added to acidify the mixture to pH 2 before extracting 6 times with 200 mL of ethyl acetate. BPPB was obtained by pooling the ethyl acetate fractions together and evaporating the solution to dryness at 45°C under vacuum.^14^

### 2.4 HPLC‐DAD fingerprinting of P biglobosa phenolics

HPLC analysis of phenolics in FPPB and BPPB was carried out as previously reported.^13^ A phenomenex C‐18 column (4.6 mm × 150 mm) packed with 5‐μm diameter particles was employed to carry out the chromatographic analyses. The mobile phase was 2% acetic acid in water (A) and methanol (B). The composition gradient was as follows: 5% of B for 2 minutes and 25%, 40%, 50%, 60%, 70%, and 100% B at 10, 20, 30, 40, 50, and 60 minutes, respectively. The phenolic extracts (free and bound) were dissolved in methanol (10 mg/mL), and the flow rate and injection volume were 0.7 mL/minute and 50 μL, respectively. Comparison of the retention time and UV absorption spectrum with those of the commercial standards allows for the identification of phenolic compounds in the extracts. Prior to use, all the samples and mobile phase were filtered through 0.45‐μm membrane filter (Millipore) and then degassed by an ultrasonic bath. Stock solutions of standards references were prepared in the HPLC mobile phase at a concentration range of 0.020 to 0.250.

### 2.5 Animals

Male albino rats (Wistar strain) weighing between 270 and 320 g were used for this study. They were kept in cages and provided food and water ad libitum and maintained in a room with controlled temperature (22°C ± 3) with 12‐h light/dark cycle. The use and maintenance of animals were in line with the guidelines of the Brazilian Association for Laboratory Animal Science.

### 2.6 Antioxidant assays

#### 2.6.1 Total antioxidant activity

The ABTS test was employed to measure the total antioxidant activity as described previously.^9^ The Trolox equivalent antioxidant capacity (TEAC) was obtained by calculating the percentage inhibition of absorbance and plotting it as a function of the concentration of standard and sample. Division of the straight line gradient of the plot for the sample by that of Trolox gave the TEAC (graphs not shown).

#### 2.6.2 DPPH radical scavenging activity

DPPH radical‐scavenging activity of P biglobosa extract and reference compound (ascorbic acid) were determined using a standard method.^9^ The ability of both FPPB and BPPB to scavenge the DPPH radical was monitored at 517 nm.

#### 2.6.3 Reducing property

The ferric reducing property of both FPPB and BPPB was determined as previously described^9^ using the synthetic antioxidant, butylated hydroxytoluene (BHT) as a reference compound.

#### 2.6.4 Anti‐hemolytic effect

The anti‐hemolytic activity of the phenolic extracts was determined as described by Yang et al.^15^ Blood (5 mL) was taken from healthy volunteers (*n* = 3) and centrifuged at 1500 rpm for 3 minutes. The resulting red blood cell pellets were washed thrice with sterile phosphate buffered saline (PBS) solution (pH 7.4) and then diluted to 20% cell suspension with PBS. To approximately 250 μL of resulting red blood cell in a test tube, 250 μL of FPPB and BPPB (0, 5, 10, 15, 20, and 25 μg/mL final concentrations) and 125 μL of HgCl_2_ (5 μM) were added. The mixture was incubated at 37°C for 3 hours in a water bath. Thereafter, 1 mL of PBS was added, and the mixture centrifuged at 2000 g for 10 minutes. The absorbance of the supernatant was read at 540 nm. For the control, the extract was replaced with PBS. Ascorbic acid (0, 10, 15, 20, and 25 μg/mL final concentrations) was used as reference standard.

### 2.7 Assay of enzymes of neurological and cardiovascular relevance

#### 2.7.1 Acetylcholinesterase (AChE) and butyrylcholinesterase (BChE) activities

Activities of acetylcholinesterase (AChE; EC 3.1.1.7) and butyrylcholinesterase (EC 3.1.1.8; BChE) were determined using the spectrophotometric method described by Ellman et al^16^ with slight modifications. Fresh whole brains of male albino rats were harvested and homogenised in 10 parts of cold 60 mM Tris‐HCl, pH 7.4 (1/10, w/v). The homogenate was centrifuged at 3000 g for 10 minutes to obtain a clear supernatant. Exactly 100 μL of sodium phosphate buffer (100 mM, pH 7.5) containing 10 mM DTNB, 10 μL of FPPB/BPPB (5, 10, 25, 50 μg/mL), and 10‐μL supernatant were added in a 96‐well microplate (SpectraMax M2e Multi‐Mode Reader, USA) and incubated for 5 minutes at 25°C. The addition of 20 μL of acetylthiocholine iodide or S‐butyrylthiocholine iodide (8 mM) initiated the reaction. The hydrolysis of the iodides was monitored at 412 nm by following the enzyme‐catalyzed formation of the yellow 5‐thio‐2‐nitrobenzoate anion, as a result of the reaction of DTNB with thiocholines.

#### 2.7.2 Na^+^/K^+^‐ATPase activity

Na^+^/K^+^‐ATPase activity was evaluated in freshly prepared whole brains of male albino rats incubated with FPPB or BPPB as described by Wyse et al.^17^ The brains were harvested and homogenised in 10 parts of cold Tris‐HCl buffer (10 mM, pH 7.4) and centrifuged (3000 g, 10 minutes) to obtain a clear supernatant. The assay mixture consisted of 50 μL of Na^+^/K^+^‐ATPase substrate buffer (pH 7.4) (containing in mM, 30 Tris‐HCl, 0.1 EDTA, 50 NaCl, 5 KCl, and 6 MgCl2), 50 μL of phenolic extract (FPPB/BPPB—5, 10, 20 μg/mL), 50 μL of supernatant (50 μg protein) with or without ouabain (1 mM, 50 μL). The addition of 50‐μL adenosine triphosphate to a final concentration of 3 mM initiated the reaction and the mixture was further incubated for 30 minutes at 37°C before terminating the reaction with 70 μL of TCA 50% (w/v). Quantification of the released inorganic phosphate (Pi) was done by using a reaction mixture containing 100 μL of ammonium molybdate (50 mM), 40 μL of the reaction mixture from first grid and 10 μL of ascorbic acid (8%). A calibration curve of inorganic phosphate was prepared using different concentrations (0, 4, 8, 10, 20, 40 nMol) of NaH_2_PO4 (1 mM). The specific Na^+^/K^+^‐ATPase activity (nmol of Pi/mg of protein/minute) was obtained by subtracting the ouabain‐insensitive activity from the overall activity (in the absence of ouabain).

#### 2.7.3 Angiotensin‐converting enzyme (ACE) inhibition assay

The assay was based on the hydrolysis of N‐hippuryl‐His‐Leu hydrate (HHL) by the ACE as described by Cushman and Cheung.^18^ The lung capillaries are the major repositories of ACE.^4^ Freshly removed rat lungs were therefore used as the enzyme source in the present study according to previous reports.^19^, ^20^ The tissue was homogenised in cold 125 mM Tris buffer, pH 8.3 (1/10, w/v) and centrifuged at 4°C for 10 minutes at 4000 g to yield a low‐speed supernatant. The reaction mixture contains 40‐μL Tris‐HCl buffer (125 mM, pH 8.3), enzyme source (50 μL), and 10‐μL phenolic extracts/drug (FPPB/BPPB—10, 25, 50 μg/mL; ramipril—0.1, 0.5, 1.0 μM). This was incubated at 37°C for 15 minutes. Thereafter, ACE substrate, HHL (8.3 mM; 150 μL), was added and further incubated for 30 minutes at the same temperature in an orbital shaker (40 rpm) before terminating the reactions with 1 M HCI (1 mL). The cleavage product of the action of ACE on HHL, hippuric acid, was extracted from the acidified solution into ethyl acetate (1 to 2 mL) by vortex mixing for 15 seconds. After centrifugation (3000 g, 5 minutes), aliquot of each ethyl acetate layer (l mL) was obtained, and the fractions were evaporated by heating at 120°C for 30 minutes. The hippuric acid so obtained was re‐dissolved in 1‐mL distilled water, and the concentration was calculated from its absorbance at 228 nm.

### 2.8 Statistical analysis

Values are expressed as mean ± SEM/SD of replicate measurements (*n* = 3). Unless otherwise stated, statistical evaluation was done using 1‐way analysis of variance followed by Dunnett's Multiple Range Test. The significance level was set at *P* < 0.05. Statistical analysis, graphing, and EC_50_/IC_50_ determinations were done using Graph Pad Prism (ver.5.0a).

## 3 RESULTS

### 3.1 Polyphenolic composition of free and bound phenolic extracts of P biglobosa

**Figure 1:**
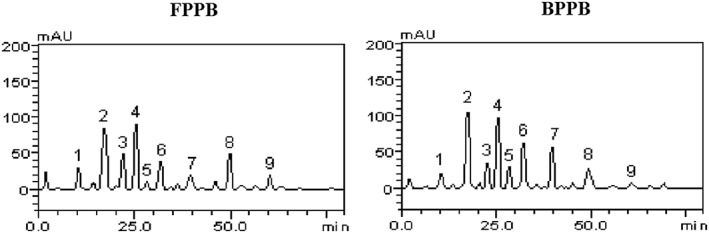
Representative high‐performance liquid chromatography profiles of Parkia biglobosa free (FPPB) and bound (BPPB) phenolic extracts. Gallic acid (peak 1), catechin (peak 2), chlorogenic acid (peak 3), caffeic acid (peak 4), epigallocatechin (peak 5), epigallocatechin gallate (peak 6), rutin (peak 7), quercetin (peak 8), and kaempferol (peak 9)

HPLC fingerprinting of free and bound phenolic extracts of P biglobosa leaves revealed that phenolic acids (gallic acid, tR = 11.78 minutes, peak 1; chlorogenic acid, tR = 22.97 minutes, peak 3; caffeic acid, tR = 25.36 minutes, peak 4), tannins (catechin, tR = 17.08 minutes, peak 2; epigallocatechin, tR = 28.67 minutes, peak 5; epigallocatechin gallate, tR = 32.05 minutes, peak 6), and flavonoids (rutin, tR = 39.83 minutes, peak 7; quercetin, tR = 48.54 minutes, peak 8; kaempferol, tR = 60.15 minutes, peak 9) are the active phenolics in both extracts (Figure 1 and Table 1). Also, catechin and caffeic acid, respectively, are the most abundant phenolics (mg/g) common to both extracts (Table 1).

**Table 1:** Phenolic acid and flavonoid compositions of free and bound phenolic extracts of Parkia biglobosa leaf

Compounds	Free Phenolics (FPPB)		Bound Phenolics	
	mg/g	%	mg/g	%
Gallic acid	10.47 ± 0.01	1.04	6.15 ± 0.02***	0.61
Catechin	29.85 ± 0.03	2.98	32.70 ± 0.01***	3.27
Chlorogenic acid	15.36 ± 0.02	1.53	11.84 ± 0.05**	1.18
Caffeic acid	30.29 ± 0.04	3.02	30.51 ± 0.03***	3.05
Epigallocatechin	2.87 ± 0.01	0.28	11.90 ± 0.04***	1.13
Epigallocatechin gallate	12.73 ± 0.02	1.27	19.65 ± 0.01***	1.96
Rutin	6.85 ± 0.01	0.68	18.17 ± 0.02***	1.81
Quercetin	17.10 ± 0.03	1.71	14.25 ± 0.03***	1.42
Kaempferol	5.53 ± 0.02	0.55	2.30 ± 0.01***	0.23

Abbreviations: BPPB, bound phenolic extract of Parkia biglobosa; FPPB, free phenolic extract of Parkia biglobosa.

Results are presented as the mean ± standard deviation of replicate measurements (*n* = 3). Data analysis was done using Student *t* test.

** *P* < 0.01,

*** *P* < 0.001 compared with the corresponding property of FPPB.

### 3.2 Reducing property, antihemolytic effect, and relative superior radical scavenging activity of P biglobosa free phenolics

**Figure 2:**
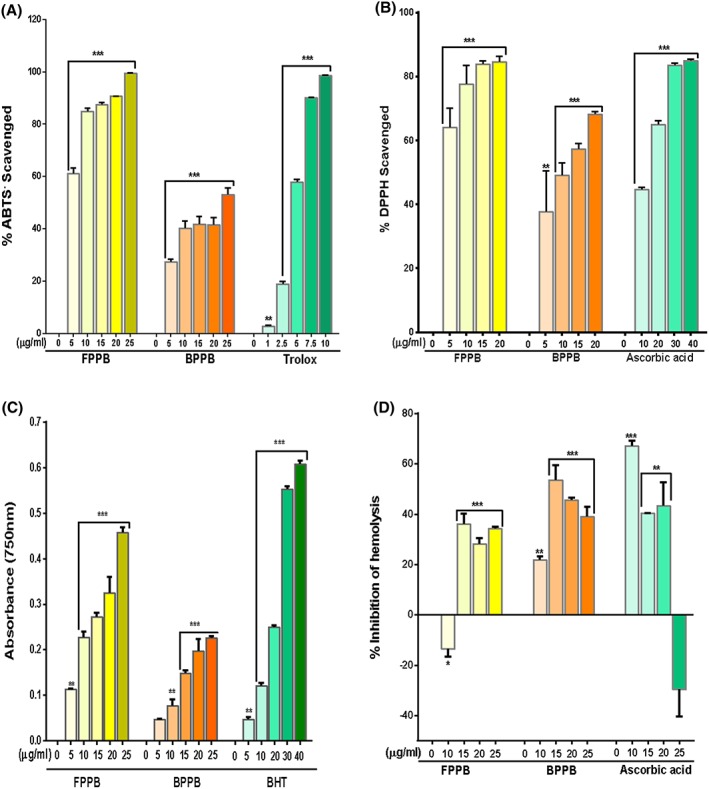
ABTS (A) and DPPH (B) radical scavenging activities, reducing property (C) and inhibition of hemolysis (D) in vitro by free and bound phenolics of Parkia biglobosa. Results are mean ± SEM of 3 parallel measurements performed in triplicates. *P* values: ** < 0.01, *** < 0.001 from 0 μg/mL FPPB/BPPB/Trolox/ascorbic acid (control). FPPB = free phenolic extract of Parkia biglobosa; BPPB = bound phenolic extract of Parkia biglobosa; BHT = butylatehydroxytoluene

FPPB and BPPB caused concentration‐dependent, significant (*P* < 0.01/*P* < 0.001) scavenging of both ABTS and DPPH radicals (Figure 2A,B). The free phenolic extract showed higher potency than the bound phenolic extract (Table 2) as reflected in their respective EC50 values for the scavenging of DPPH (3.82 ± 0.2 vs 10.22 ± 0.1 μg/mL) and ABTS (4.06 ± 0.3 vs 24.07 ± 2.1 μg/mL) radicals as well as the TEAC (0.46 ± 0.003 vs 0.23 ± 0.02 μg/mL). The ferric reducing effect of both phenolic extracts was concentration dependent (Figure 2C). FPPB (36%) and BPPB (40%) exhibited highest anti‐hemolytic potential at 15 μg/mL final concentration but with a reduction in their capacity to prevent hemolysis at higher concentrations (20 and 25 μg/mL). The anti‐hemolytic activity of the reference ascorbic acid was also statistically significant at 10 μg/mL (*P* < 0.001), 15 and 20 μg/mL (*P* < 0.01) concentrations, whereas at a higher concentration (25 μg/mL), there was a significant hemolysis of the red blood cells (Figure 2D).

**Table 2:** Trolox equivalent antioxidant capacity (TEAC) and half maximal effective concentration (EC_50_) of free and bound phenolic extracts of P biglobosa leaf for DPPH and ABTS radicals

Sample Parameter		EC_50_ (μg/mL)	TEAC
	DPPH	ABTS	
FPPB	3.82 ± 0.2	4.06 ± 0.3	0.46 ± 0.003
BPPB	10.22 ± 0.1	24.07 ± 2.1	0.23 ± 0.02
Trolox	‐	4.44 ± 0.08	‐
Ascorbic acid	12.58 ± 0.4	‐	‐

Results are mean ± SD (*n* = 3).

Abbreviations: BPPB, bound phenolic extract of Parkia biglobosa; FPPB, free phenolic extract of Parkia biglobosa.

### 3.3 Effects of FPPB and BPPB on enzymes of neurological and cardiovascular relevance

**Figure 3:**
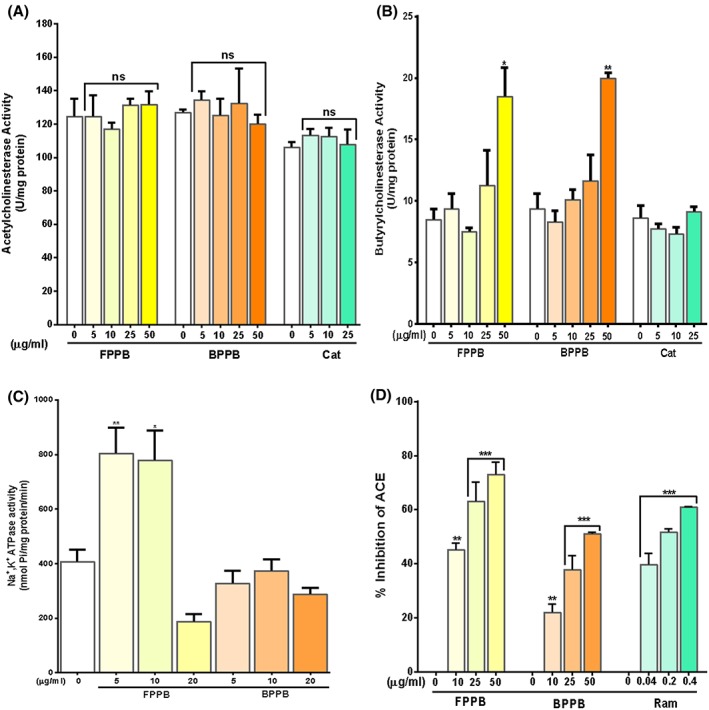
Effect of free and bound phenolics of Parkia biglobosa leaf on cerebral acetylcholinesterase (A), butyrylcholinesterase (B), Na^+^/K^+^ ATPase (C) activities and inhibition of angiotensin‐1 converting enzyme (D) by phenolics. Results are mean ± SEM of 3 parallel measurements performed in triplicates. *P* values: * < 0.05, ** < 0.01, *** < 0.0001 from 0 μg/mL FPPB/BPPB/catechin/ramipril (control). FPPB = free phenolic extract of Parkia biglobosa; BPPB = bound phenolic extract of Parkia biglobosa

FPPB, BPPB, and the reference, catechin, produced no inhibitory effect on cerebral acetylcholinesterase in vitro. Butyrylcholinesterase activity was, however, only increased at the highest concentration (50 μg/mL) evaluated for both phenolic extracts (Figure 3A,B). Increase in cerebral Na+/K+ ATPase activity produced by free phenolics of P biglobosa (Figure 3C) was significant at 5 μg/mL (97%, *P* < 0.01) and 10 μg/mL (97%, *P* < 0.01). Free and bound polyphenols of P biglobosa demonstrated dose‐dependent, significant (*P* < 0.01/*P* < 0.05) inhibition of angiotensin‐I‐converting enzyme activity in vitro (Figure 3D). As shown in Table 3, the free phenolic extract (IC50 = 15.35 ± 4.0 μg/mL) showed considerably higher inhibitory effect than the bound polyphenols (IC50 = 46.85 ± 3.3 μg/mL), but not the reference, ramipril (IC50 = 0.173 ± 0.04 μg/mL).

**Table 3:** IC_50_ values for the inhibition of angiotensin‐1 converting enzyme (ACE) in vitro

Sample	IC_50_ (μg/mL)
FPPB	15.35 ± 4.0
BPPB	46.85 ± 3.3
Ramipril	0.173 ± 0.04

Results are mean ± SD (*n* = 3).

Abbreviations: BPPB, bound phenolic extract of Parkia biglobosa; FPPB, free phenolic extract of Parkia biglobosa.

## 4 DISCUSSION

The hydroxyl groups of polyphenols often make them capable of contributing directly to antioxidative actions.^21^ Although the bioavailability of polyphenols is generally low, antioxidant phytochemicals are still able to elicit pharmacological responses because very low in vivo concentrations (nM range) are actually required for therapeutic effects.^1^, ^22^ The antioxidant and radical scavenging effects of many of the constituent phenolics of P biglobosa leaves, including catechin and caffeic acids, are well documented.^23^ Even though FPPB and BPPB are phenolic‐rich extracts, it is noteworthy that the phenolic contents of both extracts performed similarly or quantitatively better than many plant extracts reported in the literature. These include the Polish blue‐berried honeysuckle,^24^ different peach cultivars,^25^ and selected medicinal plants (*Connarus var. angustifolius*, *Cecropia obtusa*, *Cecropia palmata*, and Mansoa alliacea) evaluated by Pires et al.^26^

It could be postulated that P biglobosa phenolics reduce the stable DPPH radical to the corresponding hydrazine following reaction of the radicals with hydrogen donors in the antioxidant principle.^9^ The radical scavenging activity and reducing potential of FPPB, when compared with the reference compounds in the present study, revealed the promising profile of the free phenolics of P biglobosa as a potent antioxidant source.

The erythrocytes are among the most abundant cells in the body. Oxidative stress and erythrocyte membrane alterations may be responsible for hemolysis, with deleterious consequences.^27^ Both FPPB and BPPB could protect against HgCl_2_‐provoked lysis of human erythrocytes at a specific concentration range. The observed decrease in the antihemolytic effect of the extracts and ascorbic acid at higher concentrations could be due to the prooxidative tendency of phenolics and antioxidant molecules, including ascorbic acid, at concentrations above a particular threshold.^28^, ^29^

Phenolic compounds have been speculated to contribute towards lowering the incidence of neurodegenerative diseases.^30^ Of considerable importance to neuronal functions are the acetylcholinesterase and Na^+^/K^+^‐ATPase enzymes. Some cases of psychiatric disorders are known to involve a disruption in ion homoeostasis and are often characterised by decreased Na^+^/K^+^‐ATPase activity.^31^ In the present study, increase in cerebral Na^+^/K^+^‐ATPase activity produced by the free phenolics of P biglobosa is in line with what was obtained for the CE of the plant^13^ and might be associated with the antioxidant properties of the component phenolics, because Na^+^/K^+^‐ATPase is very sensitive to oxidative stress conditions.^32^ It was reported that prior to the formation of pathologic lesions observed in transgenic models and Alzheimer disease cases, impairment in brain function with regards to cognition and memory usually occurs as a result of oxidative stress, which induces a decrease of Na^+^/K^+^‐ATPase and other signal transduction proteins.^33^ Acetylcholinesterase and butyrylcholinesterase inhibitors are viable therapeutic targets in Alzheimer's disease, which is characterised by a cholinergic deficit.^34^ In the present study, P biglobosa phenolics caused no inhibition of cerebral acetylcholinesterase. This further supports the proposition of a different mechanism of neuroprotection possibly related, albeit partly, to the potential ability of the phenolics to prevent oxidative stress‐related damage of neurones.^13^

Angiotensin‐converting enzyme (ACE) is a zinc metallopeptidase that plays a vital role in the regulation of vascular tone. ACE functions by converting the inactive peptide angiotensin I into angiotensin II, which increases blood pressure by its vasoconstrictive effect and promotes sodium and water retention in the body.^35^ Several studies have demonstrated that phenolic compounds, isolated from different plants, can inhibit ACE activity and reduce blood pressure.^4^, ^5^, ^35^ The present study presents novel information on ACE inhibitory effect of P biglobosa phenolics. Free hydroxyl groups of phenolic compounds could chelate the active zinc ions in ACE, thereby inactivating the enzyme often through competitive inhibition.^36^ It could thus be speculated that the earlier reported hypotensive effect of the CE of P biglobosa leaf^11^ is due to the interaction of its constituent phenolics with ACE.

## 5 CONCLUSION

It is concluded that the antioxidant activity, modulation of cerebral ATPase activity, and inhibition of ACE activities by P biglobosa are related to its constituent phenolics. The latter finding could explain the biochemical rationale behind the use of the plant in the management of hypertension in traditional medicine.

## CONFLICTS OF INTEREST

There are no conflicts of interest to declare by authors.
